# A case report of prostate cancer metastasis to the stomach resembling undifferentiated-type early gastric cancer

**DOI:** 10.1186/s12876-017-0655-0

**Published:** 2017-08-07

**Authors:** Chiaki Inagaki, Takuto Suzuki, Yoshiyasu Kitagawa, Taro Hara, Taketo Yamaguchi

**Affiliations:** 1 Department of Gastroenterology, Chiba Cancer Center, 666-2, Nitona-chou, Chuo-ku, Chiba-shi, Chiba, 260-0801 Japan; 2 Hara Clinic, 228-1, Haraoka, Tomiura-cyo, Minamibousou-shi, Chiba, 299-2403 Japan; 3 Department of Frontier Science for Cancer and Chemotherapy, Osaka University Graduate School of Medicine, E21-19, 2-2, Yamadaoka, Suita, Osaka, 565-0871 Japan

**Keywords:** Case report, Gastric neoplasms, Gastrointestinal endoscopy, Prostate-specific antigen, Prostatic neoplasms

## Abstract

**Background:**

Occurrence of metastatic cancer to the stomach is rare, particularly in patients with prostate cancer. Gastric metastasis generally presents as a solitary and submucosal lesion with a central depression.

**Case presentation:**

We describe a case of gastric metastasis arising from prostate cancer, which is almost indistinguishable from the undifferentiated-type gastric cancer. A definitive diagnosis was not made until endoscopic resection. On performing both conventional and magnifying endoscopies, the lesion appeared to be slightly depressed and discolored area and it could not be distinguished from undifferentiated early gastric cancer. Biopsy from the lesion was negative for immunohistochemical staining of prostate-specific antigen, a sensitive and specific marker for prostate cancer. Thus, false initial diagnosis of an early primary gastric cancer was made and endoscopic submucosal dissection was performed. Pathological findings from the resected specimen aroused suspicion of a metastatic lesion. Consequently, immunostaining was performed. The lesion was positive for prostate-specific acid phosphatase and negative for prostate-specific antigen, cytokeratin 7, and cytokeratin 20. Accordingly, the final diagnosis was a metastatic gastric lesion originating from prostate cancer.

**Conclusion:**

In this patient, the definitive diagnosis as a metastatic lesion was difficult due to its unusual endoscopic appearance and the negative stain for prostate-specific antigen. We postulate that both of these are consequences of hormonal therapy against prostate cancer.

## Background

The prevalence of metastatic cancer to the stomach is low and ranges from 1.7% to 5.4% based on autopsy findings [1]. The most common sites of gastric metastasis are breast cancer, lung cancer, and malignant melanoma. Metastatic lesions are often more solitary than multiple occurrences and are frequently located on the greater curvature in the middle and upper third of the stomach. Endoscopically, a metastatic lesion is typically observed as a submucosal tumor with or without central depression. However, some of these metastatic lesions resemble primary gastric cancer and histological confirmation, including that by immunohistochemistry, is indispensable for differential diagnosis [2].

In prostate cancer, metastases to bones and lymph nodes are common, but metastasis to the stomach is extremely rare [3]. Prostate cancer can occasionally present as a metastatic carcinoma with unknown primary origin; however, the origin of metastasis in such a setting is easily identified by using immunohistochemistry for prostate-specific antigen (PSA) and prostate-specific acid phosphatase (PSAP).

Here, we report a case of prostate cancer metastasis to the stomach, which resembled undifferentiated-type early gastric cancer (UD-EGC), as observed on both conventional and magnifying endoscopies. Endoscopic biopsy from the lesion was negative for PSA staining and was not useful for facilitating a correct diagnosis. Caution must be applied in interpreting endoscopy findings in patients with malignancies, particularly those under treatment.

## Case presentation

The patient was a 75-year-old Japanese male who had prostate cancer with bone metastasis and high serum PSA level (7040 ng/ml, reference range < 4 ng/dL) that responded well to luteinizing hormone-releasing hormone (LH-RH) agonist for 8 months. Abdominal CT scan revealed no evidence of prostate cancer progression. He was referred to our department due to a 4-week history of epigastric discomfort. Physical examination was not remarkable. Laboratory work-up was not significant except for elevated ALP, LDH and PSA levels, which were improved compared to values before hormone therapy (Table 1).

**Table 1:** Laboratory findings before hormone therapy (A) and at referral (B)

Laboratory Test	A	B	Normal Value
WBC(×10^9^/L)	8	6	3.3-8.6
Hb(g/L)	**112**	**122**	137-168
Ht(%)	**33.6**	**36.9**	40.7-50.1
Plt(×10^9^/L)	227	183	158-348
Alb(g/L)	**39**	42	41-51
Cre(μmol/L)	**45**	**53.9**	57.4-62.7
AST(U/L)	18	27	13-30
ALT(U/L)	15	18	4-43
LDH(U/L)	**1123**	**276**	124-222
ALP(U/L)	**966**	**353**	106-322
Na(mmol/l)	140	142	138-145
K(mmol/l)	4.6	4.4	3.6-4.8
Cl(mmol/l)	108	104	101-108
PSA(ng/ml)	**7040**	**238**	<4

Serum values of LDH, ALP and PSA values were decreased with hormonal treatment. Abnormal values are given in bold type

*WBC* white blood cell count, *Hb* hemoglobin, *Ht* hematocrit, *Plt* platelet count, *Alb* albumin, *Cre* creatinine, *AST* aspartate aminotransferase, *ALT* alanine aminotransferase, *LDH* lactate dehydrogenase, *ALP* alkaline phosphatase, *Na* sodium, *K* potassium, *Cl* chloride, *PSA* prostate specific antigen

**Fig. 1:**
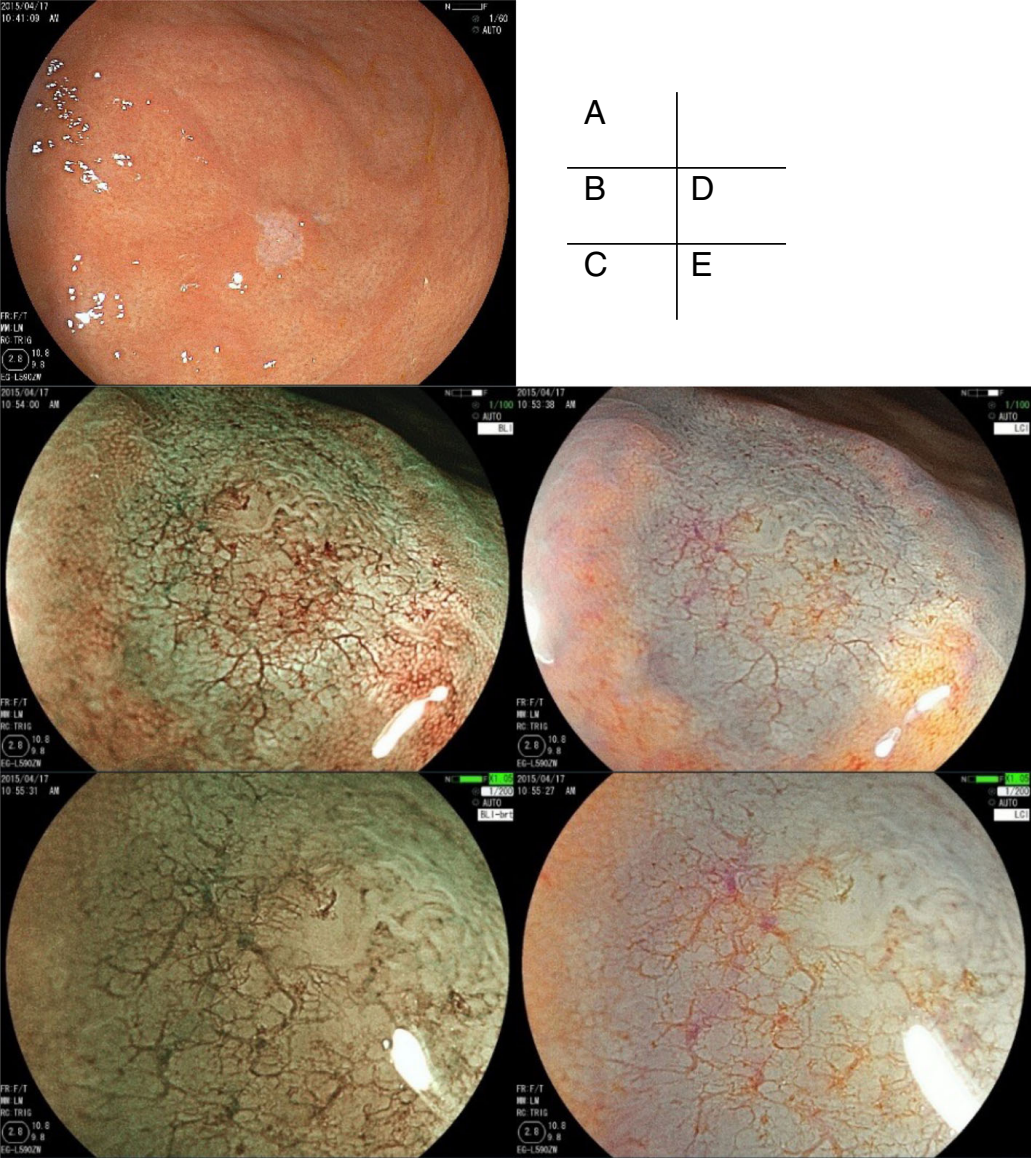
Endoscopic Findings. **a** Conventional endoscopy with WLI. A slightly depressed, discolored lesion with sharp margin was observed against non-atrophic mucosa on the anterior wall of the middle gastric body. **b**-**e** ME with BLI (**b**, **c**) and ME with LCI using indigo carmine dye spray (**d**, **e**). **c** and **e** are images with the highest power optical magnification. In the depressed area, microsurface pattern was sparse and partially absent. Microvascular pattern was irregularly irregular, that is, a variation in caliber, non-uniform shapes, and an asymmetric distribution. Both microsurface and microvascular patterns were indistinguishable from UD-EGC. WLI, white-light imaging; ME, magnifying endoscopy; BLI, *Blue* Laser Imaging; LCI, Linked Color Imaging; UD-EGC, undifferentiated early gastric cancer

**Fig. 2:**
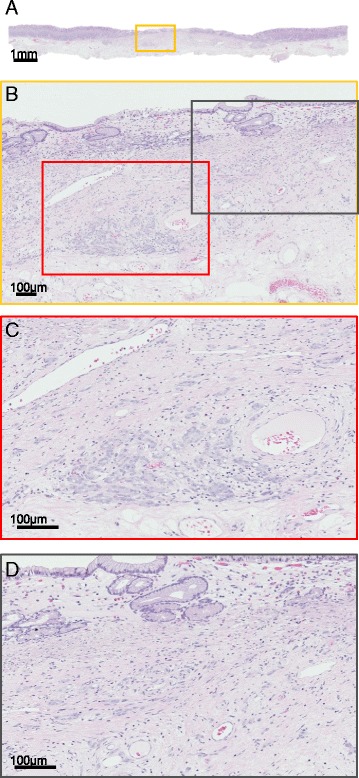
H&E staining of the resected specimen. **a** Panoramic view (×1), **b** Low-magnification view (×100) of the *yellow* frame in A, **c** High-magnification view (×200) of the *red* frame in B, **d** High-magnification view (×200) of the *gray* frame in B. Histopathological findings revealed tumor cells, which mainly resided in the superficial submucosal layer, and also showed atrophy of the gastric fundic glands as well as increased stromal tissue. H&E, hematoxylin and eosin

**Fig. 3:**
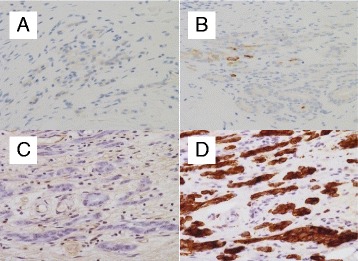
Immunohistochemical staining of the resected specimen. The tumor was negative for CK7 (**a**), CK20 (**b**), and PSA (**c**) and was positive for PSAP (**d**). CK, cytokeratin; PSA, prostate-specific antigen; PSAP, prostate-specific acid phosphatase

Esophagogastroduodenoscopy (EGD) was performed and revealed a slightly depressed, discolored lesion with sharp margin against non-atrophic mucosa on the anterior wall of the middle gastric body (Fig. 1). Magnifying endoscopy (ME) with blue laser imaging (BLI) and linked color imaging (LCI) demonstrated a sparse and partially absent microsurface pattern with irregular microvessels in the depressed area. These findings are compatible with UD-EGC. Biopsy showed moderately differentiated adenocarcinoma and immunohistochemistry with PSA was negative. Contrasted computed tomography demonstrated absence of significantly enlarged perigastric lymph nodes and also there were no new sites of metastatic disease. Thus, we initially diagnosed it as a primary early gastric cancer. Considering his prostate cancer and estimated prognosis of several years, endoscopic submucosal dissection was performed. En bloc resection was successfully achieved without complication. Histopathologic findings from the resected specimen were remarkable for moderately to poorly differentiated adenocarcinoma, which predominantly existed in the superficial layer of the submucosa. Atrophy of the gastric fundic glands, which were replaced with fibrous tissue, were observed focally near the tumor infiltration site (Fig. 2). As metastasis was suspected, immunochemical staining was performed. The tumor was negative for PSA, cytokeratin (CK) 7, CK 20, and positive for PSAP (Fig. 3). Consequently, the lesion was finally confirmed as a metastatic gastric lesion of the prostate cancer.

At the time when the pathological diagnosis of the gastric metastases was made, patient’s extragastric lesions were responding to endocrine therapy, and because of this we did not change his systemic treatment for prostate cancer.

## Discussion and conclusions

Prostate cancer metastases to the stomach is very rare. As far as we know, there are only ten cases has been reported previously (Table 2). Most of the gastric metastases were detected at the primary staging or at the time of progression. Common endoscopic features were nodules with ulceration, folds thickening and multiple ulcerations. Notably, all previous cases were positive for PSA stain.

**Table 2:** Summary of previous cases of gastric metastasis of prostate cancer

Author	Age	Treatment status at the time of gastric metastases	Endoscopic findings	IHC findings
Holderman et al. [12]	88	Naïve	Nodules with central depression, Folds thickening	PSA (+), CK (+), Mucin (−)
Christoph et al. [13]	67	Naïve	N/A	PSA (+)
Hong et al. [14]	66	Disease progression on endocrine therapy	Small elevations with ulceration	PSA (+)
Onitilo et al. [15]	89	Naïve	Folds thickening with dispensability, Ulcerations	PSA (+), CK (+), CG (−)
Onitilo et al. [15]	57	Disease progression on endocrine therapy	A broad based ulcerated exophytic lesion	PSA (+), CK (+), CG (−)
Bilici et al. [16]	69	Clinical remission with endocrine therapy	Multiple ulcerations	PSA (+), PSAP (+), CK7 (−), CK20 (−)
Mehrzad et al. [17]	71	Disease progression on chemotherapy	A nodule with ulceration	CK AE1/AE3 (+), PSA (+), CK7 (−), CK20 (−), CDX2 (−),
Soe et al. [18]	64	Withdrawing chemotherapy	Folds thickening	PSA (+), AMACR (+),
Patel et al. [19]	71	Status post surgery and radiation therapy	A nodule, ulcer and multiple erosions	PSA (+)
Bhandari et al. [20]	58	Disease progression on endocrine therapy	A nodule with ulceration	PSA (+), CK20 (+), CK7 (−),
This case	75	Responding to endocrine therapy	Slightly depressed, discolored lesion	PSA (−), PSAP (+), CK7 (−), CK20 (−),

*IHC* immunohistochemistry, *(*+) positive, (−) negative, *CK* cytokeratin, *CG* chromogranin, *PSA* prostate-specific antigen, *PSAP* prostate-specific alkaline phosphatase, *AMACR* alpha-methylacyl-coenzyme A racemase

We initially failed to achieve the correct diagnosis because of two reasons. Features of both conventional and magnifying endoscopies of our case mimic those of UD-EGC, and biopsies from the gastric lesions were negative for PSA stain.

An endoscopic examination with conventional white light imaging (WLI) demonstrated a discolored and slightly depressed lesion with clear margin, which is recognized as the typical characteristic of UD-EGC and an uncommon manifestation of metastatic stomach lesions [4]. We presume that discoloration observed on WLI is related to histological improvement, which occurs in response to hormonal treatment. Histological changes, resulting from hormonal therapy for prostate cancer, include decreased number of cancer glands and increased periglandular collagenous stroma [5]. Therefore, we hypothesized the following mechanism of discoloration. Cancer infiltration resulted in atrophy of the fundic glands. Then, in response to hormonal therapy, malignant glands disappeared and were replaced with fibrous tissue. Consequently, the mucous layer became scarce, giving rise to the discolored appearance on WLI.

Similar discoloration is observed in mucosa-associated lymphoid tissue (MALT) lymphoma at the site of tumor regression following *Helicobacter pylori* (*H.pylori*) eradication [6]. This change in color is considered to be due to a decreased number of gastric glands caused by neoplastic infiltration and elimination of lymphoid cell infiltration after *H. pylori* eradication. This histological change corresponds to our observation and may support our theory.

We also postulate that hormonal therapy contributed to the lesion’s slightly depressed appearance. In primary gastrointestinal malignancies, flattening of elevated mucosa and ulceration are observed in response to chemotherapy [7]. Considering this, slight depression of the lesion may indicate a good response against hormonal therapy and is possibly preceded by more common endoscopic pattern, e.g., a bull’s eye configuration.

On ME with BLI and LCI, we found a sparse microsurface pattern and an irregular microvessel pattern in the depressed area, which are nearly identical to those associated with UD-EGC [4, 8]. BLI and LCI are novel technologies of image-enhanced endoscopy (IEE) and considered to possess good visibility as narrow-band imaging (NBI) [9]. The utility of magnifying IEE on metastatic lesions is not well studied. In our case, observation with ME suggested U-EGC. This might occur as a consequence of histological change following hormonal treatment.

By negative staining for PSA, we reached a false initial diagnosis of primary gastric cancer for this patient. Both PSA and PSAP are highly sensitive and specific immunohistochemical markers of prostate cancer [10], which are useful for establishing the prostate origin of metastatic adenocarcinoma in diagnostic practice. However, PSA and PASA are less frequently expressed in small cell or poorly differentiated prostate carcinoma and pretreated carcinoma [5]. Unfortunately, we didn’t perform biopsy of prostate. Considerably good response to hormone therapy is incompatible to clinical feature of prostate cancer associated with aggressive histology [11]. Therefore, we suppose negative staining for PSA in this case is likely due to hormonal therapy, whereas we cannot explain why reactivity to PSAP was maintained. We only used PSA staining prior to endoscopic resection because we did not suspect metastasis based on endoscopic findings. We might have avoided unnecessary endoscopic resection if we had included additional immunohistochemical stains, such as staining for PSAP on biopsy specimen, after considering the patient’s history of treatment.

To our knowledge, we are the first to describe the case of prostate cancer metastasis to the stomach that was indistinguishable from UD-EGC. We suggest that the alterations in morphology and immunohistochemical staining owing to hormonal treatment made it a challenging diagnosis. Caution should be applied in interpreting endoscopic findings in patients with malignancies, particularly those undergoing treatment.

## Abbreviations

BLI: Blue laser imaging
CK: Cytokeratin
EGD: Esophagogastroduodenoscopy
*H.pylori*: *Helicobacter pylori*
IEE: Image-enhanced endoscopy
LCI: Linked color imaging
LH-RH: Luteinizing hormone-releasing hormone
MALT: Mucosa-associated lymphoid tissue
ME: Magnifying endoscopy
NBI: Narrow-band imaging
PSA: Prostate-specific antigen
PSAP: Prostate-specific acid phosphatase
UD-EGC: Undifferentiated-type early gastric cancer
WLI: White light imaging
